# On the Treatment of Rattlesnake Bites, with Experimental Criticisms upon the Various Remedies Now in Use

**Published:** 1861-03

**Authors:** S. Weir Mitchell


I.—*On the Treatment of Rattlesnake Bites, with Experimental Criticisms upon the Various Remedies now in Use.* By S. WEIR MITCHELL, M.D.

THE subject of the poisons made use of by certain animals has been in all times of the utmost interest to the popular mind. That it has failed to attract an equal or proportional amount of scientific investigation, can only be accounted for by supposing that the popular aversion to serpents, as well as the real danger which more or less surrounds the pursuit, have combined to deter toxicologists from engaging in such researches. The admirable effort in this direction made by the Abbé Fontana, who has left us the record of 3000 experiments on viper poisoning, may also have done something to prevent further study of this serpent, since his opinions have been reverentially received as final, and the multitude of his experiments has caused them to be looked upon as exhaustive of the subject.

In other than European countries, and where the more virulent poisonous snakes abound, observers have been wanting, or they have lacked those means of pursuing the study which only a great city affords. It has thus happened that, through want of material where observers were plenty, or lack of these where material was abundant, the knowledge of serpent venoms has advanced but little since the days of Fontana.

Before the time of that great toxicologist, viper venom had been studied by Charas, 1669, Redi, 1672 and 1675, and Mead, 1673. A multitude of others had also touched the subject, but, on the whole, they added nothing important to the information which came down from the Greek and Roman fathers of medicine; or, when they added anything, it took the shape of fanciful conjecture, and served only to make more difficult the task of unraveling the united web and woof of popular and scientific beliefs as to venomous serpents.

Without carefully reviewing this mass of strange opinions and superstitious conceptions, it is not possible to appreciate the service done by Fontana in clearing the ground for modern research and in setting at rest a host of minor absurdities. Most of the definite and novel views which he put forth as the direct results of his experiments have been more or less unsettled by various partial inquiries of more modern date: but, on the other hand, some of the most valuable facts which he discovered have never been questioned; and, as a whole, his essay, or series of essays, is still a monument of industry, ingeniously directed, and of experimental sagacity of the highest order.

From 1767, the date of his essays, no contributions of any moment were made to the toxicology of venoms until the publication of Russel on the Poisonous Serpents of India, in 1787.

In 1793 and 1799 appeared in this country Dr. Barton's Essays, which were rather records of his own thoughts and of popular and other opinions than of original research. In 1817, Mangili settled the question of the innocency of venom taken by the mouth; and in 1843, Prince Lucian Bonaparte analyzed the venom of the viper, and determined its albuminous nature. At various periods also appeared numerous papers by East Indian surgeons and European physicians on the therapeutics of snake bites; but, with trifling exceptions, no further experimental papers were produced until Drs. Brainard and Green recorded their researches in 1853. Dr. Brainard's separate Essay, 1854, contained interesting observations as to the phenomena of venom poisoning, but the main object of both the papers alluded to was the examination of the value of iodine used locally as an antidote. The tendency to regard the subject chiefly from a therapeutical point of view has indeed prevailed throughout nearly all of the researches, made either in this country, in India, or in Europe, so that if we omit the essays of Bonaparte, Mangili, Russell, and Davy, the work of Fontana still remains without a companion—no one since his time having examined any one serpent poison as to its chemistry, toxicology, and mode of formation. Yet, as every physician must concede, the treatment of snake bites can never be rationally understood until we retrace our steps and study anew and more profoundly the venom malady and its cause, in place of playing at perilous hap-hazard with its difficult therapeutics.

When I first engaged in the study of the venom of the rattlesnake, it was with the intention of ascertaining what value Bibron's antidote possessed. To effect this single end I procured four or five snakes from the Pennsylvania Alleghanies and proceeded to subject animals to their fangs, and afterward to give the supposed antidote.

After destroying many animals and attaining only negative results, I began to perceive that I was working in the dark, and that it was altogether impossible to obtain useful results without possessing definite knowledge as to the nature of the venom, the mode of its formation and ejection, and the whole natural history of the disease to which it gave rise.

The information which I desired was yet to be created. It existed in none of the books, and even so much of it as had been acquired by Fontana with regard to the viper, might not be true of the rattlesnake.

With a clear sense of these deficiencies in the present state of knowledge as to venom poisoning, I laid aside my experiments on remedies, only to resume them after the labors of two summers had removed from my path the impediments which have hitherto rendered the study of antidotes practically useless. The result of these researches is recorded at length in a paper recently published by the Smithsonian Institution, and to which I desire to refer the reader for full details of my experiments and for the conclusions to which they led me.* The principal difficulty which I encountered at the outset was the want of snakes; owing, however, to the ready and constant aid which I received from the Smithsonian Institution, I was enabled to secure a regular supply from the Virginia Alleghanies, and with these and such other chance supplies as I could purchase, or as were procured for me through the kindness of my friends, I was enabled to pursue my purpose with only such embarrassments as of necessity belong to the subject.

The practical difficulties which lay in the way of one studying the treatment of snake bites were among the most easily resolved of the many questions which multiplied in number and increased in perplexity as I advanced on this interesting path of study. In fact, I cannot but perceive that I have reopened a field of research which promises most valuable and strange results to the toxicologist, nor can I fail to comprehend that the whole subject of venom poisoning is to be reconstructed, and that on no branch of science are we so utterly ignorant as on this one. M. Bernard alone, of all the recent writers, seems to be aware of our lack of knowledge in this direction, and strongly urges a re-examination of the principal animal poisons, such as the venom of toads† and serpents.

At the close of the Smithsonian Essay, just referred to, I have given a brief statement of my views as to antidotes, and as to the great difficulties attendant upon their thorough study, and I have appended a short discussion of the relative value of the various remedial means now in repute

* Researches upon the Venom of the Rattlesnake: with an Investigation of the Anatomy and Physiology of the Organs concerned, by S. Weir Mitchell, M.D., Lecturer on Physiology in the Philadelphia Medical Association. Jan. 1861. Washington: Smithsonian Institution. New York: D. Appleton & Co. Accepted for publication, July 1, 1860.

† Medical Times and Gazette, September 29th, 1860, p. 296. M. Bernard makes some interesting remarks on the venom of the toad. M. Gratiolet had already examined this subject and arrived at somewhat similar conclusions, to which, however, M. Bernard does not allude. See Gratiolet. Comptes Rendus, vol. xxxiv. p. 731, 1851.

for the treatment of snake bites, as well as my own opinion on the rational method of treating these injuries.

The object of this present essay is to consider all the best-known antidotes by the light of the practical criticism of experiment, and, finally, to point out what means of treatment appear to me best calculated to relieve the sufferers from these dreaded accidents.

The course of study thus laid down will involve an examination of the following points, which I shall consider at such length as my space permits:—

1. Fallacies in regard to the use of antidotes of all kinds, arising from want of exact knowledge as to the secretion of venom, and the mode in which the serpent uses its fangs and ejects the poison.

2. Fallacies as to antidotes, arising from want of information on the natural history of the disease caused by the venom.

3. General considerations as to antidotes, and as to the mode of conducting researches in this direction so as to avoid errors.

4. Description of the phenomena of rattlesnake bites, analysis of symptoms, etc.

5. Local treatment. Experimental examination of the local medication most in repute.

6. General or constitutional treatment. Experimental examination of the principal constitutional remedies.

7. Sketch of the author's views as to treatment, local and general.

1. *Fallacies in regard to the use of antidotes of all kinds, arising from want of exact knowledge as to the secretion of venom, and the mode in which the serpent uses its fangs and ejects the poison.* When an antidote has been given, or any treatment used after a snake has bitten a man or a lower animal, it is usually taken for granted that the danger of any two bites is much the same if the subjects of the bites are alike in age or vigor. Now, even when the serpents are themselves of equal bulk and have at disposal drop for drop the same amount of venom, it may chance that the danger of the two bites is utterly unequal, and thus that in one an antidote might fail, and in the other appear to succeed. This arises from one of the following reasons:—

The snake fails to elevate its fangs sufficiently when striking, and the fang points touching the skin are driven backward toward their usual position of repose without penetrating the part aimed at. When this accident occurs, no wound is inflicted unless the teeth of the lower jaw be-become entangled in the skin of the bitten part, in which case the small wounds thus made may be easily mistaken for fang marks. When experimenting with Bibron's antidote, in July, 1859, a large dog was secured and placed within reach of a snake which struck it fiercely and became fastened for a short time, so that I was able to perceive that the fangs were doubled backward, their anterior convexities resting against the skin to which the serpent was attached by the curved teeth of its lower jaw. The wounds made by these teeth were of course harmless, and the dog experienced no further inconvenience. This cause of failure in the bite must be difficult of detection, under ordinary circumstances, the snake being free, since it would be dangerous to approach closely, and since the snake is usually entangled for but a brief period. When, however, the serpents are held by the middle, in a leathern loop at the end of a staff, and thus allowed to bite, they not unfrequently fail to elevate the fangs sufficiently, and, as in experiments on antidotes, it is often necessary to secure the serpents in the manner described, the possibility of this occurrence should not be overlooked.

When the rattlesnake bites, whether it be at perfect freedom or not, both fangs do not always pierce the skin of the animal stricken. I have sometimes suspected that the serpent does not always elevate both fangs. This, however, is a point which does not readily admit of direct observation in snakes at liberty, and I can only add, that of seven dogs bitten by serpents at freedom, four had two fang marks, and three had but one. Now as the fang, duct, and gland of one side are quite distinct from those of the other, if only one fang be used, the dose of poison administered will be but one-half of that which would be injected were both fangs employed.

Apart from the possibility of the snake using only one fang at will, there are other facts in this connection which may enable us to explain the frequent occurrence of single fang marks. When, for example, the snake strikes obliquely at the flank of an animal, one fang sometimes remains out of reach of the part penetrated by the other, and this is the more apt to occur, because, in elevating the fang teeth, at the moment of attack, their extremities are made to diverge widely.* For a like reason, when the serpent strikes a small limb or member, it sometimes chances that the fangs either straddle the part completely, or that one fang entering it, the other passes it to one side without in any way injuring its tissues.

Besides these cases of single fang marks, many instances occur in which, although both fangs penetrate the opposing tissues, only one is in reality

* The object of this seems to be to protect the lower jaw from injury in case the fangs miss their aim and are driven downward, in which case they would pierce the lower lip of the snake were it not that their divergence throws their points outside of it.

active, or, both entering the flesh, for reasons to be presently detailed a part, or perhaps in some cases the whole of the venom fails to be injected, and the danger of the wound is materially lessened.

When the fangs in biting are fixed in the flesh, the lower jaw of the serpent is pressed upward against the part bitten, and at the same instant the temporal muscles, and especially the anterior temporal, compress the venom gland, and urge its accumulated venom along the duct and through the tooth. In most cases where both fangs have been used, the actions which bury the fangs more deeply, and inject the poison, are consentaneous on both sides; but sometimes a perceptible interval appears between the contraction of the right and left sets of muscles, so that a sudden motion of the bitten animal occasionally liberates one fang before its charge of venom has been duly delivered.

Still more curious, however, is it, that we may have both fangs deeply buried in the flesh of an animal, and yet not a drop of venom injected. The explanation of this source of fallacy in the use of remedial means is to be found in the following facts. Redi, in his researches on viper venom, published in 1675, states that the poison passes down alongside of the fang, and between it and the mucous cloak which covers it when at rest, and which is now known as the vagina dentis. Fontana disproved this statement, showing that the venom passes out through the canal of the fang. If, as I presume is the case, the arrangement of which I shall presently speak belongs to the viper as well as the rattlesnake, both were right and both were wrong. Professor Christopher Johnston, of Baltimore, and Professor Jeffries Wyman have both of them recently described the venom duct of the rattlesnake as ending in a papilla, which projects *into* the basal aperture at the base of the fang. Upon close inspection it can be seen that no tissues connect these two parts together in any direct manner, the end of the duct being held in close contact with the fang by the gum, which envelops the tooth, and through which the extremity of the duct passes. When the fang is couched, at rest, in its mucous sheath, the apposition of the fang and duct is still kept up, but is less perfect than when the fang is erect, since then the mucous cloak is thrown off the anterior convexity of the fang, and gathering in firm folds at the base of the tooth, firmly presses the papillary end of the duct into the lower orifice of the fang.

Such being the case, it can be seen that if the fang is not fully erected, or if, from any cause, the end of the duct is separated from the fang opening, a part or the whole of the venom may escape between the fang and its mucous cloak, and fall innocuous on the skin of the bitten animal. In a modified form this result often happens, and a part at least of the poison is cast on the skin, the larger portion traversing the duct, and probably the excess alone being wasted. In direct experiments on animals I have often noted the escape of venom alongside of the fang, and in general, the more the serpent's motions are interfered with during the experiment, the more likely is it that the whole or a part of the poison will be lost in the way I have mentioned. It thus happens that the most vigorous serpent may become innocent at the very moment of the bite, and that not even the most watchful attention will enable the observer to say that the remedy given was the cause of a bite proving mild in its effects. As I have here urged, this, like other sources of fallacy, is most apt to appear when the serpent is held, and when thus we endeavor to cause the bite to occur in a particular part of the body of an animal.

2. *Fallacies as to the value of antidotes, arising from want of information in regard to the natural history of the disease caused by the venom of serpents.* There exists an idea, not confined to the popular mind, that the bite of the rattlesnake is an extremely fatal accident. Although we have no full statistics which are available to settle the matter, I have gathered enough information from various sources to enable me to assert with great confidence that it is far less fatal than has been supposed. When making this statement, I do not mean to be understood as saying that the rattlesnake is not a dangerous animal, but only that neither man nor dog need be regarded as condemned to death when wounded by it, whether remedial means are afterward employed or not. A large rattlesnake long restrained from biting will use his weapons no doubt with deadly effect, and hence, when showmen have been bitten, they have rarely escaped. On the other hand, the greater number of such accidents, arising from serpents at freedom, will be apt to prove serious in their results, but not very often fatal. Of fifty-seven cases of rattlesnake bites which are given in full or merely mentioned in the journals, only five died; and even if we make every allowance for the character of the reports, this evidence still remains sufficiently strong; nor have I found that it lost force in the presence of such facts as my experiments on animals have brought before me. A close analysis of the table of cases in my Smithsonian essay, (p. 100,) with reference to the treatment and the result, brings us to the conclusion either that all treatment (oil, alcohol, iodine, ammonia, etc.) is successful, or else that the greater part of the cases must have survived under any form of medication. It can be shown, moreover, that most of the plans of treatment employed are utterly useless.

Here, then, is a malady from which at least seven-eighths of the patients recover. The mere fact of their surviving can assuredly be no test of the value of a plan of treatment. Yet, that this or that case did not die *has*

been thus construed, and this cardinal error exists in almost all of the earlier examinations of antidotes, and in some of the later ones.

Authors who have reported successful cases of the treatment of snake bites by various means, have been further misled by a want of knowledge as to the duration of cases not treated at all, and as to the character of the recovery. A general survey of a number of cases, and a careful study of animals bitten and not treated, can alone supply this lack of information as to the average natural history of cases undisturbed by any therapeutic resorts. As the result of such study, we learn that a few cases of rattlesnake bite die, that a few linger long ere recovery is complete, and that the larger proportion get well, and that with a degree of suddenness which is sufficiently surprising, considered with reference to the serious character of the symptoms, and well calculated to deceive the credulous therapeutist.

In many cases this abrupt departure of all serious symptoms is most remarkable; a man is bitten, thought to be dying, treated this or that way, and on horseback or at work within forty-eight hours from the time of the bite. In dogs bitten, alike result obtains, and the recovery after the most urgent symptoms is usually rapid and complete.

Now nothing is more gratifying to the physician than the sudden effect of his remedies; and the speedy and favorable change of a case from an appearance of extreme danger to one of relief and convalescence, naturally leads him to attribute that result to his medication, which really was natural to the malady. A fuller acquaintance with the disease must annihilate this source of error for any but the most incautious minds.

3. It will now be fitting to consider the third section of our subject, and to comprehend clearly how we may avoid the sources of fallacy above pointed out, and *how the study of antidotes to serpent poison should be conducted.*

And first, what is an antidote? The popular mind usually conceives of it as a remedy having power to neutralize directly in the system a given poison, destroying its potency by acting upon it chemically or otherwise, in some more mysterious way. It is possible that agents of this kind may exist, but thus far we are ignorant of any possessing such a relation to the venom of the rattlesnake. The pretensions of remedies supposed to be so gifted may be easily settled by mingling them with the venom, and afterward injecting the mixture into the tissues of an animal.

The more rational conception of an antidote, is of an agent which merely counteracts the effects of the poison, and which may have no chemical influence on the poison itself. Such an antidote may enjoy no power to affect the toxic activity of venom when mixed with it, and yet may prove to be an active constitutional preservative against its effects. Just this position seems to be held by one of the supposed antidotes most in repute.

So far as I am aware, no great difficulty is likely to arise in the study of antidotes, owing to their nature as such, for, although some of those most in esteem, such as Bibron's antidote (bromine) and the Tanjore pill, (arsenic,) are poisonous in a high degree, it is easy to learn how much may be given with impunity. Hence, although in one or two instances observers have actually and plainly destroyed animals with the very agents which were designed to relieve them, it is not probable that errors of this kind will be perpetuated, or even occur so often as to bring into disrepute any really useful remedy. The mode in which the study of antidotes and local remedies should be conducted, so as to avoid all the fallacies to which I have alluded above, will now claim our attention.

Opportunities of studying the use of remedies, local and general, in connection with cases of venom poisoning in man, are of course more or less rare, and it is scarcely possible to eliminate or allow for the varied fallacies which surround with difficulty this method of studying the subject. Owing to this, and to the comparative ease with which the means of study may be created in animals, it is preferable to employ these, and to use only such treatment in human cases as may appear to promise enough of success to justify its usage.

The larger the animal employed the better will it be for the purpose, since the symptoms are more easily studied in large animals, and since they are less likely to be injured by active antidotes. Almost all toxicologists who have investigated this subject, have been content to submit animals, as dogs, etc., to be bitten by the serpents themselves. We have seen, however, that when this course is followed, a number of fallacies interfere to prevent the observer from drawing satisfactory conclusions, and although great care and thorough acquaintance with the anatomy and habits of the serpent may enable us to overcome this difficulty in part, some portion of the obstacles in question are in the nature of things unavoidable. In my own researches I have sought to escape from these embarrassments: first, by a careful study of the natural history of venom poisoning in dogs and other animals; second, by injecting into the animal experimented upon known quantities of venom previously removed from the ducts of active serpents. The venom to be thus employed is secured in the following manner: A serpent is seized by the middle, with a leathern loop at the end of a staff; then the neck is caught and held down on a table with a notched stick, while a tube an inch and a half in diameter, and holding a sponge soaked in chloroform, is slipped over the snake's head, and by a dexterous motion carried downward so as to include one-third of the length of the serpent, the notched stick being at the same time removed. About twenty minutes are required to stupefy the snake. It is then seized by the neck, and the edge of a saucer slipped under the upper jaw, so as to elevate the fangs. This is done by an assistant while the operator with his right thumb and forefinger strips forward the glands and ducts on both sides. The yellow venom runs out through the fang and alongside of this weapon. A known amount of this fluid may then be injected into the tissues of an animal, the instrument employed being a minute trocar and syringe. It may be objected to this method of using the venom, that it is supposed by many persons that the poison is less fatal when used artificially than when injected by the snake. Of this, however, there is no adequate proof, and I have seen nothing to induce me to believe that it is at all correct. On the other hand, the advantages arising from the artificial use of the venom are manifold and obvious, and it is only essential to know what amount of venom is certain to destroy a dog if no remedial agency intervenes. If, in addition to this, the observer is thoroughly cognizant of the ordinary phenomena of the venom disease, he will possess all the reasonable means of insuring accuracy, which are now attainable. Antidotes may then be used internally, in one of two ways to be hereafter illustrated, or mingled with the venom, and injected where this mode of study is to be desired.

Before stating my experiments upon the plans of treatment now or recently in repute, it will be proper to give the reader certain necessary information as to the nature of venom poisoning, the forms it affects, and the symptoms which characterize its varieties. These details must of necessity be brief, and the reader who wishes more complete information is referred to the author's previous paper.

The venom of the rattlesnake is a yellow, albuminous fluid, of an acid reaction, of a sp. gr. of 1044, and coagulable at a temperature of 140° to 160°. Its toxic activity is unaffected, or but slightly affected by boiling, and not at all by freezing. Acids and alkalies, alcohol, etc. do not destroy its virulence, and when dried it retains its dreaded power for an unlimited period of time. Closer qualitative analysis discovers in it at least two albuminous substances: one coagulable by boiling, either when alone or diluted with water, and also by alcohol; the other, also albuminoid, coagulable by alcohol only, and constituting the active element of venom. This latter agent I have described as *crotaline*.

*Effects of venom on man and animals.*—When an animal receives in any way a dose of venom, one of two things happens. If the animal is small, or at all events if relatively to the size of the animal the amount of venom injected is large, the animal dies very suddenly, acutely poisoned. If, on the other hand, the dose of venom is relatively small, the animal suffers to some extent with the symptoms of acute poisoning, and then passes into what I shall term the stage of chronic or secondary poisoning, which may endure for an indefinite period, and end in death or recovery. Acute poisoning in man is rare,* and is more and more common the smaller the bulk of the animal bitten, until we arrive at cold-blooded creatures, in whom this sudden ending is the exception, and great prolongation of the malady (*i.e.* secondary or chronic poisoning) the rule. In dogs I have rarely seen the very rapid death I speak of; but it is not uncommon where the serpents are large and active and their venom abundant.

Let it be clearly understood then, that when man or animal is poisoned by venom, a set of symptoms occur which wind up with death, or, being prolonged, pass into others of a somewhat different nature, constituting the chronic cases, or those which survive long enough to exhibit the signs which characterize the secondary poisoning.

When, for example, a pigeon is bitten, or receives in any way three or four drops of venom, it walks a few steps, crouches, gasps for breath, rolls over, and is dead in a few minutes, convulsed or not in the moment of agony. So sudden and speedy is this ending in some cases, that the pigeon may die within a minute. The only additional symptoms which we can perceive are the rapidly quickened and enfeebled motions of the heart, and sometimes vomiting and evacuations from the cloaca. In larger animals the same symptoms take place, but the vomiting is more common and the expression of general debility more perceptible.

Men who have been bitten describe their symptoms as much the same in kind, but, as before stated, they rarely end in death in this stage at least; the power of resistance acquired by increased bulk being, I presume, the chief protective agency. In some cases the more formidable signs of prostration do not declare themselves before some minutes or even half an hour has passed. In one case a man engaged in splitting wood was bitten; he picked up a stick and pursued the snake a few feet, when suddenly he became sick at the stomach, complained of deadly nausea and general weakness, reeled a few steps farther, and fell on the ground. In another instance, the sufferer walked briskly for twenty minutes before the symptoms of debility became very well marked.

It becomes important to our purpose to decide the cause of these symptoms and what organs are affected. All authors agree in speaking of the condition as one of debility, and all describe the pale face and cold sweats, the hurried breath and quick and feeble pulse. If we

* Though rare, not impossible; men have died from this cause within twenty minutes of the time of the bite, although no such cases are on record in the journals, and are only known to me by personal information.

examine an animal dying rapidly with these symptoms, we find absolutely no lesions—the blood and the tissues are alike healthy in appearance—both to the naked and assisted eye. A series of experiments, the relation of which would be misplaced here, has shown that the heart does really become enfeebled, and that the arterial pressure is singularly diminished; and this appears to be a direct effect on the arterial system, since it is impossible long to sustain life by artificial respiration; at the same time the nerve centres are attacked, and the respiratory movements failing on this account, become jerking and labored; the sensory and motor nerves seeming still to preserve their functional integrity. Such, in general terms, I suppose to be the causation of death in these cases. Far different are the symptoms which arise for study when the patient survives the stage of acute poisoning. The duration of this stage it is indeed difficult to define; this only we know, that after a time the debility continuing, as shown by vomiting and syncope, the blood becomes affected in a marked and singular manner, while the relations of tissue and fluid are so altered that passive hemorrhages take place; jaundice occurs, and a variety of symptoms declare themselves as this or that organ becomes diseased and the seat of congestion and ecchymosis. Meanwhile the local symptoms assume an importance which they do not possess in the acute stage, and may even become of paramount influence in deciding the fate of the patient.

If then the patient die very early, there are symptoms of weakness alone, and there are no perceptible lesions of blood or tissue; supposing life to be prolonged, the early symptoms continue, while signs of blood poisoning appear in addition, and lesions closely resembling those of yellow fever are found post-mortem. If, again, the patient successfully resists the secondary evils here described, he may still perish from the results of the local injury, which increases in danger and importance as the case progresses.

To make this matter clear we will now examine more accurately the various symptoms and the character of the wound.

*Wound.*—The wound is usually described as very painful, but so far as my own experience informs me it is not always so in animals, nor do all men who are bitten speak of it as painful at first. Indeed, the wound has sometimes been for awhile disregarded, and at all events the hooked form of the fang, the forcible injection, and the sudden withdrawal of the weapon, account sufficiently for the pain, without supposing it to be specific. The succeeding local symptoms are rarely notable when the patient dies within half an hour, except that in animals the muscles twitch most violently, of which we hear nothing in the human cases. As the case advances, the part swells, becomes discolored and increasingly painful, and these changes extend up the limb involved, and, reaching the trunk, swell and bloat one side, or the whole body.

This swelling is not inflammatory, but arises from the gradual effusion of blood, which has lost power to coagulate, and which therefore extends from the broken vessels, at the seat of the wound. The later swelling is also due more to œdema than inflammation, although it seems probable that in man the tendency to inflammation under venom poisoning is greater than in the lower animals. In dogs bitten, the local swelling is sometimes slight, sometimes enormous, and when cut into is found to depend on a collection of blood, either fluid or semi-coagulated. The pain which accompanies the swelling is excruciating in many cases, and does not lessen until the part becomes vesicated, loses heat and falls into gangrene In man this process destroys the skin only, or the whole of a limb, but in dogs I have seen no such extensive sloughs, and the skin often escapes, so that we find only a small opening, and beneath it a cavity containing the débris of broken-down tissues, mixed with pus. I suspect that in man the swelling would occur less rapidly were it not for the constant use of the ligature about or above the wounded part. If the case be a serious one, the early constitutional signs of prostration continue; occasional vomiting, or at least nausea, is present, frequent syncope occurs, and the pulse continues weak and rapid. In general the bowels are constipated, unless the case be greatly prolonged, when diarrhœa may take place as a sequel. The mind is, in most instances, clear up to the time of death, or at all events it is only confused, excited, or subject to sensory delusions, while convulsions seem to be of extreme rarity in any period of the malady in man. The fall of temperature in the skin is usually described as an early symptom of the general weakness, but no accurate thermometric observations have been made in human cases. The duration of cases of rattlesnake bite is very various, although both in dogs and men the recoveries are often rapid and unexpected.

As it is impossible for me to dwell in full detail upon the symptoms of the venom malady, and equally impossible to describe the great variety of lesions which may occur, I have thought best to state three typical cases of poisoning in animals, and two in men. The following are quoted in full from the essay so often referred to:—

"*Experiment.*—The dog, a small terrier weighing about fifteen pounds, was intended to make one of a set of observations on the value of Bibron's antidote. For this purpose he was placed in the snake-box, where instantly he was struck twice by a large snake, both wounds being double fang marks, and both being in the right flank. On removing him I observed that from one of the wounds blood was running in a thin stream. After it had run for some time, I caught a few drops in a watch-glass, and found that it coagulated well. Before I thought fit to use the supposed antidote, I was called away. Returning at the end of an hour I found the dog standing with his head pendent, having just vomited glairy mucus. His pulse was quick and feeble, his respiration occasionally panting. The hemorrhage had ceased. Owing to an accident which at this time deprived me of the supply of Bibron's antidote, which I had prepared, I was unable to employ the animal in the manner proposed, and not desiring to lose the observation altogether, I made use of the opportunity in the following way:—

"One hour and a half after he was bitten I drew a drachm of blood from the jugular vein. It clotted perfectly.

"Four and a half hours after the bite a drachm of blood from the same vein coagulated equally well.

"Twenty hours from the time of the poisoning, the dog was found lying on his left side, having passed slimy and bloody stools in abundance. At intervals he seemed to suffer much from tenesmus, but was so weak that he stood up with difficulty. His gums were bleeding, a symptom I had seen before, and his eyes were deeply injected. At this time about two or three drachms of blood were drawn. It was very dark, and formed within five minutes a clot of feeble texture.

"Twenty-seven hours and a half after the time at which he was bitten, the dog was weaker. His hind legs were twitching, and the dysentery continued. Three drachms of blood were drawn as usual, but no clot formed in this specimen although it was set aside and carefully watched for some time. While I was collecting the fluid for observation the dog suddenly discharged per anum at least four ounces of dark, grumous blood. At this time I supplied the dog with water, and left him. Fifty-four hours after the bite he was seen again, and found to have drunk freely of water, and to have passed fewer stools. Up to this date he declined all food.

"From this time he improved rapidly, and took with eagerness whatever nutriment was offered. On the fourth day his blood again exhibited a clot, although it was very small and of loose texture. I made no further examinations of the blood. The dog lost flesh as he gained strength, and had profuse suppuration from an abscess in the bitten flank. At the close of two weeks he was active and well, except that the wound was still open.

"The case last related is doubly valuable, as pointing out even in a single instance the time at which the blood became altered, and also as showing, once more, how profound may be this change, and how perfect the recovery.

"*Experiment.*—A dog of mongrel bull-terrier breed, weighing thirtyone pounds, was lowered into the cage, where he was struck on the outside of the right hind leg in the thigh. He drew up the leg when released, and whined for a few minutes. The wound, which was a double fang mark, bled a drop or two, and the muscles about it twitched considerably at intervals for an hour, when this symptom was obscured by the swelling. His pulse, which was naturally about 145 and irregular, was, at the fifth minute, 140 and regular, respiration 35. At the fifteenth minute he lay down, much weakened, pulse 160 and feeble, respiration 40. At the twentieth minute the bowels moved loosely, with a gray discharge, and there seemed to be some tenesmus in the rectum. Twenty-fifth minute, pupils so far natural and mobile; he could stand when urged, but lay down again at once, and was much weaker. Forty-fifth minute, pulse 160, respiration 45 and laborious. Fifty-fifth minute, loss of power in the hind legs. Eightieth minute, respiration quick and labored, and so irregular as to make it impossible longer to count the heart pulses. The eyes were natural, and followed my motions; and he wagged his tail when fondled. At this time the observation was temporarily interrupted, and, on its resumption at the third hour, the dog was found dead. He had no foam about his mouth, and probably died quietly.

"*Post-mortem section.*—The whole muscular and areolar tissue of the leg and thigh, half way up and down the limb, was dark with infiltrated blood. About the wound the swelling was due to a mass of blood partially coagulated. The extravasated blood extended through the limb, and on the inside it passed half way up the sartorius and adductors, and along the sheath of the vessels to within two inches of the femoral ring. Nearly an inch of the sheath was clear of it, but one-half inch below the ring the tissues were shaded with blood, and the same appearance was seen around the ring itself. From this point the extravasation extended under the peritoneum, into the pelvis, and on to the inner face of the ilium. The color of the tissues thus stained was a brilliant scarlet. The abdominal viscera were healthy, except that the mucous membrane of the lower bowels was somewhat congested. The lungs were sound. The heart was relaxed, the right side full, the left nearly empty. The blood on the right side was a little darker than that on the left; on both sides and everywhere else it was perfectly fluid and free from clots. Placed in a phial, it remained fluid until decomposition ensued. Two hours after death, some of the blood globules found in the heart were slightly indented; those taken from the small vessels of the ear were perfectly normal. At the period of examination, the muscular and nervous irritability had entirely departed."

A third observation on a smaller animal, and also drawn from a like source, will answer my present purpose.

"*Experiment.*—In this instance the animal, a rabbit, was struck once in the back by a large snake already exhausted by frequent use. A few minutes after the bite took place, the rabbit was seized with weakness, gritting of the teeth, and rapid respiration. It passed urine and feces, and remained feeble during some hours. From this period the weakness abated somewhat, but the back continued to swell. On the second day the local signs were improving, but the animal had passed a very albuminous urine, and a large amount of blood mixed with feces. The symptoms of general weakness now increased, the hind legs began to drag, the motions were uncertain, and the bloody purging grew worse. The rabbit died on the third day, during my absence.

"*Post-mortem section.*—Rigor well developed. The period of death being uncertain, the irritability of the tissues was not tested. The wound was surrounded by half an ounce or more of dark fluid blood. The vessels in the neighborhood were full of a similar fluid, but there was no vascular redness, like that of acute inflammation. The muscles in the track of the bite, which was a double fang mark, were remarkably softened and could be torn with the utmost ease. The brain was highly congested, and there was a good deal of bloody serum in the cavities of that organ. Similar congestion existed in the spinal canal, and at several points the white nervous tissue was stained with small patches of blood. The lungs were healthy. The pericardium was curiously distended with bloody serum. The heart was contracted and contained but little blood, and that dark and diffluent. The intestines were spotted at intervals with ecchymoses four to five lines in diameter and apparently just beneath the serous covering, the cavity of which contained a little bloody serum. The intestines from the œsophagus to the rectum were dotted with ecchymoses and filled, especially the large gut, with blood and mucus. The right kidney was large and absolutely soaked with dark fluid blood. The left kidney was more healthy. The bladder and ureters contained a good deal of bloody urine. How the rabbit lived so long with such a singular complication of serious lesions it is difficult to conceive. In most cases of chronic poisoning, some one or two organs may become the seat of local extravasations, but for extent and character of lesion this case stands alone in my experience."

The accounts given by our own authors of the cases of human poisoning are extremely meagre and unsatisfactory—the best reports being those by Sir E. Home* and M. Pihorel,† both of whom described cases which took place in showmen.

---

* Engl. Phil. Trans. at large, 1810, p. 75.

† Journ. de Phys. Exp. et Pathol., vol. viii. p. 97.

Dr. Horner's* case is perhaps one of the best among our own reports, but like the more curious one given by Sigaud,† the patient was not altogether a healthy subject. I shall quote Dr. Horner's case with the statement that the local symptoms were better illustrated in the second case quoted, that by Dr. R. Harlan.‡

---

* Am Journ. Med. Sci., vol. viii. 397, 1831.

† Du Climat et des Maladies de Brésil, p. 394, contains the following account of the singular case above mentioned, a case so curious in every point of view that I have been tempted to add a translation of it.

"According to a popular idea in divers parts of America the bite of the rattlesnake cures the leprosy (lèpre léontine of Alibert) without injury to the patient. Many facts would seem to show that lepers have been bitten without fatal results, not only by the coraline viper, by the jararacasu, but even by the rattlesnake. Among these facts may be cited those which have been collected by Dr. Jacintho Pereira Reis, and by the deputy, Estevaõ Rafael de Carvalho. The first is that of a leper of the district of Rio-das-Velhas, in the province of Minas-Geraes, who having been bitten by a rattlesnake, was cured of his disease in fifteen days. The second is furnished by a negro slave of the province of Maranhaó, who recovered from his leprosy in a very short time after having been bitten. M. de Lima assures us that being one day in the town of Saint Charles, province of Carabobo, in Colombia, he observed a man whose face was covered with a single large cicatrix, which at first he attributed to a burn, but on inquiry learned that it was the result of a radical cure of a case of leprosy, by the bite of the rattlesnake. The same observer tells us that an opinion favorable to this means of cure is entertained throughout the district of Caracas and Apure, where leprosy is common. These statements which, after all, are but hearsay, induced a leper to resort to this fearful mode of relief. According to Dr. Jacintho Rodrigues Pereira Reis, another leper had already made this experiment in this capital, (Rio de Janeiro.) This person had the courage to allow himself to be bitten at one time by the coraline viper, and at another by the jararaca preguicosa. Each time he was left for dead, but notwithstanding, gradually revived without aid. After this he still had the pain to observe that his original malady continued its fearful progress.

"*Case.*—Marianno Jose Machado, born at Rio Pardo, province of Rio-Grande do Sud, aged fifty, had been for six years afflicted with tubercular lepra. During four years he had resided at the leper hospital, at Rio de Janeiro. On the third of September, he came out, resolute to put to the test the bite of the rattlesnake, despite the prudent and wise counsels of divers physicians, who saw in the means he desired to employ a more than dubious chance of success, and who also were aware that the patient had not exhausted all the more available and proper remedies. The patient finally resorted to the house of M. Santas, a surgeon, Rue de Vallongo, No. 61, who possessed a rattlesnake." After describing the appearance and character of the leprosy, and mentioning those who were present, M. Sigaud continues as follows:—

"Marianno Jose Machado, before proceeding, declared that he acted on his own responsibility, and then having signed a paper to this effect, put his hand into the

‡ North Amer. Med. and Surg. Journ., xi. 227, 1831.

"Adam Lake, aged about forty, a robust, muscular man, acting in a laborious capacity, and who, from his own account, was in the habit of

---

cage and twice seized the serpent. The reptile at first fled, and finally licked his hand, but, feeling itself pinched with force, turned and bit him at the metacarpal articulation of the little finger and ring finger. The bite took place at 10 minutes of 12, September 4th. The patient did not feel the bite, and only knew of it by the remarks of those around him. His hand was a little swollen, but painless, and bled somewhat, the pulse and respiration remaining normal. Five minutes later, he experienced a slight sensation of cold in the hand, with a little pain in the palm, which, in a few minutes, increased considerably. 17th minute, pain in the wrist. 20th minute, hand swelling. 30th m., pulse stronger and fuller, mind tranquil. 55th m., sensation of swelling in sides and back of neck; size of hand increasing; pain extends to two-thirds of the forearm. 59th m., general numbness. 1 hour and 20 minutes, general tremor; hyperæsthesia. 1 h. 36 m., mind troubled, pulse more frequent; difficulty in moving the lips; tendency to sleep; choking sensation; intense pain in the hand and whole arm; hand swelling. 1 h. 45 m., pain in tongue and pharynx, extending to the stomach; increased pain and swelling in the bitten hand; feet cold. 2 h. 5 m., difficulty of speech, and a little later, difficulty of swallowing; anxiety; copious sweating on the chest. 3 h., weakness; nose bleeding; inquietude; pulse 96. 3 h. 4 m., general sweat, and a little after, involuntary groans; pulse 100; great pain in the arm; face injected; continual epistaxis. 3 h. 35 m., the patient swallows wine and water readily, and changes his shirt; a red color is seen throughout the body, and a little blood leaks out of one of the pustules under the arm; the color deepens, especially in the bitten limb; atrocious pains are felt incessantly in the arms: the throat seems to be narrowed, and the breathing becomes difficult. At 4 h. 30 m., pulse 104; salivation; great heat of body. 5 h. 30 m., pulse 104; torpor; urine abundant; saliva thick; muscular weakness; groans from excessive pain; respiration tranquil; pulse full; increased swelling of the bitten hand. 7 h., somnolence; awaking, complains of great pain in the chest, and of a sense of constriction in the throat; free and full urinations: deglutition more difficult; saliva abundant; continued epistaxis; entire inability to swallow. 8 h., inquietude; copious urination. 9 h. 15 m., profound sleep. 10 h., patient took three teaspoonfuls of infusion of guaco; refusing sugar and water which were also offered; the epistaxis now ceased; pulse 108, regular; the leprous tubercles on the face and arms are a little depressed, and have an erysipelatous look. 10 h. 20 m., made two ounces of clear urine; better; sleep for a few moments; pain in the chest lessened, and pain is now felt in the legs and feet, which with the bitten hand are still cold; pulse 108, regular; thirst; the patient drinks water; sitting up with facility. At 11 h., takes four spoonfuls of strong infusion of guaco. 11 h. 45 m., urinated a colored urine; continues to drink without trouble; pulse 119; arm and hand much inflamed, with excessive pain. 12 h., sleep; excitement; urination. 12 h. 30 m., sleep; anxious face; cries of pain; the patient demands the last offices of his church, and refuses remedies. Rather later, emission of urine; great heat in the limbs; the patient takes two doses of the remedy at successive half hours; symptoms as before. 14 h., sits up twice, to drink water; the difficulty of swallowing augmenting. 14 h. 13 m., takes the remedy; sleeps; pulse 110. 15 h. 30 m., micturates; sleeps. 15 h. 45 m., takes a dose of the remedy; involuntary movements of the right hand and left leg. 16 h.

drinking from half a pint to a pint of alcoholic liquors daily; on Friday, July 1, 1831, was in a crowd collected at Fisher's tavern, in Kensington, to witness a popular exhibition of rattlesnakes, confined in a cage. Lake being somewhat intoxicated, opened the door of the cage, and allowed one of the animals to creep out and ascend his bare arm; as it was going up, he caught the animal somewhat abruptly by the neck, it immediately struck at him, and inflicted two small wounds. In the evening, he felt some itching about the bend of his arm, and he rubbed it accordingly, without thinking of the snake. The itching increasing, he was induced to examine the part, and there he found a little red spot. The recollection of the rattlesnake then occurred to him, and he began to bathe the part in salt water. This not relieving him, he called upon Dr. Elkinton, at which time the whole extremity was swollen to nearly double its size, and was very painful. Dr. Elkinton applied a dry cup over the part which had originally itched, and was bitten; it was near the cephalic vein at the bend of the arm; scarified cupping was also done in three or four places in the same region, and some ounces of blood were extracted by a repetition of the cups; the forearm was also rubbed with the terebinthinate tincture of cantharides, which produced vesication. In the course of the evening, some doses of spirit of hartshorn were administered, and also some tablespoonfuls of the expressed juice of plantain, (*alisma plantago,*) and hoarhound, (*marubium vulgare.*)

"The next morning (July 2, 1831,) the patient was brought to the almshouse, about half-past eight o'clock. He had vomited in the conveyance. He was sensible, and stated that the scarifications had bled much during the night; they were then bleeding freely. The arm, from the shoulder and front of the thorax to the fingers, was swollen to twice its natural size, and was very painful when moved. His pulse was almost imperceptible and thread-like, his extremities cold, he was disposed to cramp in the legs, and his debility very great. His respiration was natural and easy. His eyes were muddy and heavy; his face was somewhat bloated.

---

45 m., takes a spoonful of the remedy; repose; pulse 100; two emissions of urine during 17th and 18th hours; respiration being easy. 21 h. 45 m., great prostration; convulsive movements of jaw and lower extremities; bloody urine. 22 h., pulse quickened and absent at long intervals; increase of convulsive movements; diminution of swelling of extremities and of the dark color of the skin; deglutition very difficult; breathing labored; blisters were applied to the thighs, and the infusion of guaco given. 22 h. 50., convulsive; motionless; an injection of brandy given. 22 h. 55 m, convulsions stopped. 23 h., same condition; an ounce of oil of lézard given by the mouth; was taken with great difficulty. Death, at 23 h. 30 m. The corpse became livid, and swelled considerably in a few hours, being mottled with violet-colored spots. The odor was such, next day, as to forbid an examination post-mortem."

Feeling the desire to go to stool, he was assisted from his bed for that purpose, but was seized, while on his way, with a general spasm, without foaming at the mouth; being laid down on the floor of the ward, it went off in a few minutes, and he there had an involuntary evacuation from the bowels, of a dark bilious color. This occurred before I saw him. He received from the resident physician five grains of ammonia and an ounce and a half ol. olivarum.

"Sinapisms were also applied to his ankles and breast; he was directed to take liquor volat ammoniæ, ʒj; sp. vin. dilut. ℥ss, every two hours, and intermediately use ol. olivarum, ℥j; of the former prescription, he took two doses before he died, and one of the oil. Another application of cups over the old scarified parts was made, and the hemorrhage from them diminished. The extremity was then enveloped in cloths, dipped into ice water.

"The symptoms continued stationary till 11½ A.M., he then complained of violent pain in the course of the colon, and, on taking his last dose of medicine, he said he felt sleepy, closed his eyes, and in a few minutes died without agony or convulsion.

"*Dr. Harlan's case.*—On Monday, the 13th of September, 1830, Daniel Steel, a showman of living animals, in this city, was severely bitten by a large male rattlesnake, immediately below and on the metacarpal joint of the index finger of the left hand; the accident occurred about four o'clock P.M., on a warm day, while he incautiously seized the reptile by the neck, not so close to the head but that the animal was able to turn upon him. Immediately after the bite, the blood flowed freely from both the fang punctures; the parts in the immediate vicinity of the punctures became tumid and livid, notwithstanding the efforts of the patient at suction with his mouth—which faintness obliged him soon to relinquish. On my arrival, about half an hour after the accident, I found him extremely pale and faint, and was informed that he had fainted several times, the whole of the back of the hand was puffy and tumid, with infused non-coagulated blood, which appeared to have infiltrated from the vessels and forced its way through the cellular tissue; a ligature had been previously applied on the wrist; another was now placed on the arm, the forearm having already commenced swelling.

"The situation of the wound rendered the use of cups inapplicable, and the flow of blood was so rapid as to make their application inexpedient. The punctures were separated some distance from each other, which rendered it requisite to excise two large portions of integument; the excisions extending down to the tendinous fascia; the blood, which flowed freely after the operation, did not appear disposed to coagulate; cold water was now poured on the wounds in a continued stream, from the mouth of a pitcher, held at a considerable elevation, and the swollen parts in the vicinity of the wounds were forcibly pressed, in order to expel the effused blood. The patient again became very faint, and was laid in a recumbent posture. The wounds were next washed with spirit of hartshorn, several doses of which were administered internally; but being now informed that the patient had drunk freely of sweet oil, the hartshorn was omitted, until the stomach should be evacuated by drinking warm water. A poultice of bread and water was next applied, to encourage the bleeding, and the patient was put to bed. At ten o'clock P.M., I was sent for in haste; the patient was thought, by attendants, to be dying. The bleeding of the wounds had been extensive, the tumefaction had extended up to the arm, the inner and inferior portions of which were discolored by effused blood; the patient vomited incessantly; he complained of insatiable thirst, and drank cold water every few minutes; he had pain and stricture at the pit of the stomach, great restlessness and anxiety, cold skin, with the exception of the wounded arm, which was very painful; add to which, there existed delirium, singultus, difficulty of breathing, and pulse at the wrist scarcely perceptible. The poultice, bandages, and all ligatures were immediately removed; the back of the hand was blacker and more swollen, and the skin of the forearm was hot and tense. As a substitute for the poultice, and in order to suppress the bleeding, which appeared to endanger the life of the patient by the debility it occasioned, large flat pieces of fresh meat, were bound on the wounds, hand, and forearm. Before this operation was completed, the patient exclaimed, 'That feels comfortable.' The indications arising from the present symptoms, were: 1. To allay irritation and thirst. 2. To arrest the vomiting. 3. To procure sleep, if possible. 4. To excite the sanguineous system to resist the depressing power of the poison, which had so emphatically manifested itself on the system in general.

"A mustard plaster was directed to be applied to the pit of the stomach; sixty drops of laudanum to be administered every half hour, until the vomiting should be arrested; after which the following bolus, to be taken every two hours until sleep should be induced: ℞ pulv. opii, six grains; pulv. gum. camph., eighteen grains; pulv. carb. ammonia, thirty grains. M. ft. in three boluses. Sig. as directed. Of these pills he took three before the effects desired were manifested. On the morning of the second day, his pulse was raised; the extreme thirst and irritability of the stomach were allayed, and reaction of the system in several respects was manifested; but the tumefaction of the arm had extended to the shoulder, with broad black streaks up to the axilla; stricture at the breast and great local pain were now the chief complaints. The application of raw meat was renewed, as it afforded comfort to the patient, and appeared to reduce the swelling of the hand, and by pressure, had nearly suppressed the hemorrhage. In order to allay the pain and tension of the whole arm, he was directed to expose it naked to the *fumes of burnt wool*, in a convenient apparatus, which was attended by such marked alleviation of symptoms, that the patient himself was desirous to have the operation frequently repeated, and continued for two or three days; the swelling always diminishing after each application; it caused the arm to perspire profusely, and covered it with blackish soot impregnated with ammonia, resulting from the decomposition of the wool. During the intervals, the arm was rubbed with volatile liniment. The raw meat having become offensive from its disposition to ferment and putrefy, was omitted, and flaxseed poultices substituted; the anodyne boluses were continued in half doses through the day, and the quantity increased at night to produce sleep. The system again became depressed and appeared to struggle with the effects of the poison; as the patient had been somewhat addicted to intemperance, he was allowed milk-punch to support his strength. On the third day, a greater degree of reaction was obvious; the bowels were evacuated by castor-oil; the dose of the anodyne was diminished, and by carefully nursing the arm, in less than a week suppuration supervened, and the patient was able to leave his bed."

It is much to be regretted that physicians in this country should have paid so little attention to the venom malady as only to report cases in which they supposed themselves to have been successful. Hence is it that no good history of the disease can be made out from their statements, and hence it is that post-mortem examinations of the lesions are almost unknown—there being only three on record, of which two took place in Europe. The reader need not be surprised then at the small amount of accurate knowledge of symptoms placed at the author's disposal: fortunately the ability to create the disease in animals enables us in some measure to fill up this gap.

The information now in possession of the reader will enable him, I trust, to follow the remarks upon the use of remedies. A number of these, whether local or constitutional, may be readily dismissed, either because their value or want of value is plain, or else because former observers have settled their therapeutic position in some conclusive manner.

In treating of this matter, I have been obliged to deny notice to a host of herbs which enjoy repute in small sections of our country, and which, in turn, have once possessed and lost a wider reputation. The reader who calls to mind what has here been said as to the many fallacies which surround the observer, will not fail to perceive in the accounts of these remedies given by authors the reason of their apparent success and ultimate loss of favor.

*Local Therapeutics.*—Following the classification adopted in my essay, we may divide the various local means of treatment into four classes:—

1. Agents which remove the poison and the poisoned part, as excision and amputation.

2. Means which partially remove the venom, or more or less detain it in the injured part, as scarification, ligature, suction, and caustics.

3. Agents, which being applied to the wounded part, or injected into it, are supposed to destroy the venom, or render it in some way innocuous, as injections of iodine.

4. Local usage by inunction or otherwise, of various substances, such as alcohol, indigo, ammonia, olive oil, simple warm or cold poultices, water dressings, etc.

Excision, or amputation of a small part or member, has been occasionally resorted to, the former by Dr. Harlan, the latter in a successful case which took place in France soon after M. Pihorel's case. Dr. Harlan's patient was extremely ill, but recovered.

Class 1st. The experiments of Fontana on viper poisoning have shown conclusively that immediate excision or amputation of the part bitten will save the life of an animal which must otherwise have died, and however swiftly the poison may pass into the circulation, it does certainly seem clear that in a severe case the removal by these decisive means of even a part of the venom might favorably determine the balanced chances. The question of resort to these ultimate surgical means in cases of poisoning in man, is one involving many considerations. Thus, where it is known or probable that the serpent was a large one, and where both fangs entered, and where the early symptoms were grave, free excision, or even amputation—of a finger, for instance—would be justifiable. While on the other hand, where the reverse of these circumstances is met with, or information as to the size of the snake, etc. is lacking, it would be well to remember how few fatal cases occur, and to accept for the patient the chances of a less heroic local treatment. It should also be recollected that the value of ablation lessens as we recede from the time of the original injury, unless the circulation has been arrested by ligature or cups immediately after the infliction of the wound. The means just discussed depend for their use on certain very simple considerations, and require at our hands no experimental questioning.

In class 2d we have, first, scarification or incision, which, like ablation, is of some use if done early, and is justifiable later in the case because it is so much more mild a resort. When used at all, the track of the fang wounds should be opened by the knife, and the part afterward exhausted of blood, and as far as may be of venom, by the use of suction, cups, or pressure. Caustics are more doubtful remedies in these cases than has been supposed. Potassa, soda, ammonia, and the undiluted mineral acids affect very little the toxic activity of the venom when mixed with it for a time, unless heat is also employed in addition. The actual cautery is more efficient, as it destroys both venom and tissues, and is more likely to be within reach. Like all the other local means, it is less useful as time advances. It has been but little used in this country.

*Ligatures, suction, cups.*—One of the oldest remedies in snake bites is the ligature, the simplest, most ready, and for a time the most effective means. Whenever the bite is on an extremity, a cord or handkerchief should be tied tightly about the limb, as near to the wound as possible. After a time, the swelling will necessitate its removal, when a second ligature should be placed on the part, a little higher up, and this ready means of quarantining the poison should in no case be abandoned until such necessary local treatment as seems requisite shall have been instituted, and the proper constitutional remedies employed. If it is possible, as it sometimes is, to use both the cupping-glass and the ligature, it will of course be best to do so; but cups alone can be employed in certain localities, and in some, as the nose, neither of the means at present under review can be made available, and suction by the mouth of a bystander may be the only resort; unless preceded by incision, or unless the fang mark is large enough to bleed spontaneously, this latter means is not likely to be of much service in removing the venom.

*Intermittent ligature.*—Ligation obtains for the patient a reprieve, of which the physician may make excellent use. A period arrives, however, when the local swelling threatens gangrene, and the band being then of necessity loosened, the venom rapidly overwhelms the system. Are there no means of securing the advantages of the ligature, without subjecting the sufferer to all the inconveniences of its employment, as commonly advised?

About twenty years ago two physicians of Charleston, South Carolina, Drs. Holbrook and Ogier, used the ligature in a way which was then novel. They loosened the band for a few minutes at a time, and then tightened it again, as the system began to show signs of feebleness. By this means, patiently followed out, they succeeded in admitting to the general circulation such small doses of the poison at any one time as to diminish materially the ultimate danger of the bite in the animals which they employed.* Dr. Alexander,† (1855,) in a short but very sensible essay ad-

* These experiments were made, as Dr. Holbrook informs me, by one of his pupils, superintended by Dr. Ogier. They have never yet been published.

† St. Louis Med. and Surg. Journ., vol. xiii. p. 116, 1855.

vised a like use of the ligature, and urged also that alcoholic stimulants should be given before relaxing the ligature, and afterward in such quantities and at such intervals as may seem desirable. So far as I am aware Drs. Ogier and Holbrook first experimented on this use of the ligature, and Dr. Alexander first advocated it in print, and reported cases of its use in man in combination with the internal administration of stimulants. Dr. Alexander relates the following case in illustration of his views.

The bite was in a child, near the tendo achillis. The limb was corded below the knee, and whisky being freely given, profound intoxication followed its use. "Regarding this as an indication of safety, I announced to the parents, at the end of sixteen hours from the reception of the bite, that the child was out of danger, and directed the removal of the cord. At this time there was intense swelling and commencing vesication below the cord, but not a trace of poison above it. Immediately after the removal of the cord the swelling advanced up the limb, as it had done previous to its arrest by the cord; the poison reached the trunk, as could be perceived by the swollen condition of that side of the body when compared with the other, and in two hours after the announcement of my joyful prognosis the heart ceased to beat. My patient was dead."

Dr. Alexander's further remarks, which fully represent the author's views, are as follows: "Thus instructed by this unfortunate case, I never afterward administered alcoholic stimulus until I was ready to remove the cord, which should not be done for four or six hours, or at least until the cups have effected all that they can accomplish. It should then be merely loosened, and the whisky or brandy given, so as to keep the pulse natural or full. And I would here observe that intoxication should be avoided on account of the debility which it induces; a moderate exhilaration is all that is necessary. In one or two hours more I loosen the cord still further, continuing the use of the spirits, and thus by admitting the poison into the circulation gradually, I counteract its influence more successfully than if I were to allow the whole amount of poison that the veins were capable of absorbing, to assail the heart at once."

Class 3d, of local remedies, consists of various substances such as alcohol, olive oil, ammonia, indigo, and the numberless herb infusions, poultices, raw meat dressings, etc., which at different periods have found favor in this or other countries.

If alcohol and ammonia have any value, it is purely as local stimulants. The advantages of alcoholic agents used internally, and the supposed value of ammonia similarly employed, have led, however, to the idea that they would prove specifically useful as local antidotes. As we shall see in future, the utility of stimulants taken by the mouth depends less upon their meeting the poison and neutralizing it than upon their power to counteract its physiological effects on the heart and nerve centres, and perhaps also on the blood. Moreover, the following direct experiments prove with sufficient clearness that these agents in no way directly destroy the toxic activity of venom.

*Experiment.*—Two drops of venom and ten drops of alcohol were left in contact for ten minutes, and then injected into the breast of a pigeon. It died in 71 minutes.

*Experiment.*—Two drops of venom and twelve of alcohol were left in contact for half an hour, and then thrown under the skin of a pigeon. It died in 62 minutes.

*Experiment.*—Three drops of venom and forty of alcohol were mingled, and after twenty minutes injected under the dorsal skin of a rabbit. It died in 37 minutes.

Six similar experiments ended in the same way, all the animals—three pigeons and three rabbits—dying within various periods. As test experiments, I injected small amounts of alcohol into animals of these same species, but except more or less stupefaction, observed no serious effect. In another series, two or three drops of venom were placed in a drachm of alcohol and the alcohol afterward evaporated slowly. The poison which remained was then slightly diluted with water, and injected as usual; but in each case with the ordinary fatal effect. Under these circumstances, it is plain that if, when directly mixed with venom, alcohol does not destroy its activity, it is little likely to do so when merely placed upon the wound. The experiments with ammonia as a local antidote, were made in the same way and with like results. Only one difference was perceptible: the local extravasation and swelling were not so well marked when alcohol and venom were injected together, as when venom alone was thus employed; while in regard to ammonia, I could not perceive this modification of the local phenomena. Inunction with oil was once a favorite remedial means, and was studied by Fontana with reference to viper venom, and by him pronounced useless. I have made no experiments upon its employment, nor do I suppose that any physician is likely at present to displace for it more potent and plainly indicated remedies.

The remaining local resorts enumerated above may equally be dismissed with the briefest notice. Poultices of various kinds, meat dressings, etc. are probably of some value in the later stages of the local treatment, where such applications are usually needed to aid in softening and removing gangrenous parts. I have not considered them as worthy of serious attention under any other point of view; and the same may be said of a multitude of herb applications of which Dr. B. S. Barton gives a long list, and to which the credulity of sixty years has added considerably.

Most of these have been used internally and externally at the same time, and have enjoyed a reputation which can only be explained by reference to the general considerations in regard to constitutional remedies which have already been urged upon the attention of the reader.

Class 4th, *injection of iodine in solution*.—In 1853, Dr. David Brainard, of Chicago, in conjunction with Dr. Green,* proposed the sub-cuticular injection of an iodized solution of iodide of potassium as a local antidote in rattlesnake bites. He further desired to extend this treatment so as to include poisoning by woorara, which he believed owed its toxic power to a serpent venom, an opinion general at that time, but now no longer held.

Dr. Brainard's process is as follows: Ten grains of iodine and thirty grains of iodide of potassium are dissolved in an ounce of water. The bitten part is first cupped, or a ligature is placed on the limb, until the tissues are so swollen with serum as to allow of the injection passing readily through the distended areolar spaces. A small trocar and canula is then pushed laterally into the bitten part, so as to reach the site of the wound, and the injection effected by screwing to the trocar a small syringe charged with the iodine, and so filling the part by pressing down the piston of the syringe while the cupping-glass remains over the wound and exhaustion is kept up with its aid. Apart from the antidotal value of this ingenious means, it is clear that the necessary apparatus is rarely at hand, and that cups of various curves, to fit the equally various surfaces of the body, as advised by Dr. Brainard, are not likely to come into general use in localities where the rattlesnake is found.

Before passing to the practical examination of this remedy, it is proper to state that Dr. Brainard describes it as a direct antidote enjoying the power to neutralize the venom. He adds:—

"My experiments were commenced with the venom of the rattlesnake, and made principally on pigeons, dogs, and cats. Birds die from it sooner than quadrupeds, and are therefore much more difficult to save by treatment. The way of using the serpent is, to have it confined in a groove, with only the head projecting. Denude the breast of the pigeon of its feathers, and when the fangs are raised in anger, press it against them. I prefer this method to that of extracting the venom and inoculating it, for two reasons:—

"First. The poison, when extracted, is uncertain in its operation, being often composed in part of saliva, and sometimes wholly of that fluid.

* Comptes Rendus de l'Academie des Sciences, 1853, p. 811. Brainard and Green. See also an 8vo. pamphlet, Chicago, 1854, by Dr. Brainard; and Smithsonian Reports, 1854.

Fontana constantly found that the wounds made by inoculating the venom were less dangerous than the bite of the viper.

"Second. The bite is the wound we are called upon to treat, and is placed in circumstances less favorable than a wound by inoculation."

One-half of the pigeons bitten and thus treated, died. The serpent employed was the *crotalophorus tergeminus*,* or prairie rattlesnake.

The method of using the serpent, described by Dr. Brainard, is open to objections already stated. The presumed mixture of the venom with saliva could scarcely occur, as the duct of the venom gland serves for it alone, and under no circumstances is it mixed with saliva unless the two fluids accidentally meet in the serpent's mouth. It is certainly true that Fontana found inoculation less effectual than the bite, but it is to be remembered that he in nowise imitated the mechanism of the bite when inoculating,† so that it is hardly fair to compare the results of the two processes. A fine trocar and syringe enables us to simulate the fang, and yet to use known quantities of venom. With these few remarks, we will pass at once to the experimental mode of criticism. It was clear that if animals bitten could be saved by subsequent injection of venom, and if iodine possessed the power of neutralizing the venom, a mixture of iodine with the venom ought to render it altogether innocuous when injected. At the same time we should thus escape from the numerous fallacies which embarrass all the other modes of study in which the antidote is used *after* the injury has been inflicted.

*Experiment.*—About six drops of venom were divided into equal parts. One-half was injected into the breast of a pigeon, which fell in 6 minutes, and in 80 died convulsed. The remaining half was mixed with 30 drops of the iodine antidote, and after 3 minutes the whole was thrown into the breast of a pigeon. At the close of 90 minutes the pigeon was crouching and very feeble; 27 hours later it appeared well. The local swelling was solid and not large, nor did it afterward slough or suppurate.

*Experiment.*—About 15 drops of venom were divided into three portions. Five drops injected into the breast of a pigeon killed it in 6½ minutes.

*Experiment.*—Five drops of venom were mixed with 10 drops of the iodine solution, and, after 1 minute, injected into the breast of a pigeon. Death took place in 32 minutes with convulsions

*Experiment.*—Five drops of the same venom were mingled with 1

---

* Or, more properly, Crotalus tergeminus.

† His inoculations were made on the surface of wounds, or by pushing the venom into incisions previously made.

drachm of iodine solution, and, after 3 minutes, the mixture was injected as usual into the breast of a pigeon. At the 12th minute general convulsions took place; and death followed at the 17th minute.

*Experiment.*—About 12 drops of venom from one snake were separated into two parts. About one-half was treated with 1 drachm of iodine solution. At the end of 6 minutes the mixture was injected into a pigeon's breast. At the 4th minute the pigeon fell—death occurring at the close of 85 minutes.

*Experiment.*—Three and a half drops of venom and 35 drops of iodine solution having remained in contact 23 minutes, were injected into the thigh of a pigeon. At the 5th minute it fell, and died without convulsions at the 20th minute.

*Experiment.*—Three and a half drops of venom were mixed with one drachm of iodine solution, and after 43 minutes the whole was injected into the thigh and breast of a pigeon. In 20 minutes it fell, and died in 73 minutes, not convulsed.

A series of five experiments of like nature was made on pigeons, using for each case 3 drops of venom and 25 of iodine solution. No. 1 died in 24 m.; No. 2 and No. 3, in 18 m.; No. 4, in 95 m.; and No. 5, in 127 m. In a comparative series where no iodine was used with the venom, No. 1 died in 7 m.; No. 2, in 16 m.; No. 3, in 23 m.; No. 4, in 49 m.; and No. 5, in 71 m.

A comparison of these results and a reference to the above stated experiments, suffice to show that while iodine seemed to retard the constitutional action of the venom, it did not annul that action. I am therefore unable to confirm Professor Brainard's views. If it be urged that I did not use cups in addition to the antidote, I would reply that my desire was to test the value of this antidote apart from any interfering aid, such as cups, whose utility we already comprehend. If, moreover, the cups were used by Dr. Brainard only to detain the venom in the part, and so secure to the antidote the chance of meeting it, this is needless where the venom and iodine are mixed before using them.

There remains for examination another fact of great value, first observed by Dr. Brainard. In all the cases in which Dr. Brainard injected iodine into the site of the fang wound, he found a remarkable absence of the usual local phenomena, such as swelling, ecchymosis, hemorrhage, etc., and he explained this deficiency by supposing that the venom was altered specifically by the antidote, and had thus lost its power to produce either its local or general effects. So far as the absence of the usual local signs is concerned, I can fully corroborate this statement. If venom alone be injected in one case, and venom and iodine in another, the difference between the two wounds will be such as to impress the most casual observer—the one being black, swollen, and dripping with blood; the other showing slight change of color from the iodine, but not otherwise altered.

The question now arises, is the altered character of the wound due to the influence of iodine on the venom, or to its effects on the tissues? To settle this point, I added the iodine solution as usual to portions of venom, and then exposed them to a gentle heat for two or three hours, hoping to drive off the iodine. In this I was but partially successful; only a part was thus lost, and, at all events, the iodide of potassium still remained. Thus prepared, the venom was injected. The local signs which followed were much more marked than before, and led me to suppose that if I could entirely dissipate the iodine, I should restore to the wound its original character. It would have been possible to separate the venom and the antidote by following the process of M. Reynoso,* whose admirable experimental criticism upon iodine as an antidote to woorara is a model of such research. This means, however, involved exposure of the venom to other chemical agents, and the end in view was attainable by simpler means.

After observing the local influence of iodine, I was led to employ simple astringent agents in the hope of finding that they possessed equally with this substance the power of modifying the local action of venom. A series of experiments was therefore instituted, using tannic acid in place of iodine. On mixing this agent with venom and injecting the mixture, I found that the local effects of the wound were equally well counteracted, and that, so far as lessening the danger of the wound was concerned, tannic acid answered as well as iodine. Pursuing the matter still further, I treated the venom with tannic acid in excess, and then added ammonia until the previous precipitate was redissolved. The whole mixture was next injected, and the animals were found to die as usual with signs of local and constitutional poisoning. It was also observed that although these agents affected the local phenomena, the usual signs of blood poisoning took place when the animals lived long enough to allow of this occurring. From these data, I drew the general inference that both iodine and tannic acid delay the action of the venom, by affecting the tissues rather than the venom.

Notwithstanding this conclusion, there remains the unmistakable fact that these agents so modify the local phenomena as to lessen the ultimate danger, and that, moreover, unlike caustics, their use does not involve any loss of tissue, so that they are conservative of the part, and in so far valuable and available where circumstances admit of their employ-

* Comptes Rendus, xxxix. 67, and xl. 118, 825, 1153.

ment. In a practical point of view, it is of little moment whether they effect this desirable result by action on the venom or the tissues.

In Dr. Brainard's experiments, pigeons were employed, and, in following him, I have naturally used them also. Death took place, however, so invariably when I used full doses of venom, that I could obtain but one chance of observing whether the wound continued to preserve a normal appearance, or whether in spite of this, the secondary local results, such as gangrene, etc., would occur. To satisfy myself as to this, I made the following experiments:—

*Experiment.*—A rabbit received in his flank one-half drop of venom, mixed with 18 of iodine solution. He became feeble in 10 minutes, but rallied, and finally recovered; at the 30th hour he was killed with woorara, and the wound examined. The tissues about the wound were a little gray, and shrunken, but not otherwise changed, and there was no effusion of the black blood, such as usually gives peculiarity to the wounds made by the venom alone.

*Experiment.*—Three drops of venom and sixty of iodine solution were injected into the lower part of the thigh of a dog, weighing twenty-nine pounds. The animal was ill for several hours, with feeble pulse, labored respiration and vomiting; next day he was well, but without appetite. He was killed by pithing, and the wound examined. The muscles were a little altered in color, by the iodine; but except in one locality—about two inches from the trocar wound—there was no ecchymosed appearance, and even in this spot it was trivial.

It usually happens in dogs bitten, that, on recovering, the locality of the wound forms an abscess, filled with bloody pus, and the débris of neighboring tissues, but not involving any considerable loss of skin. In two additional cases of dogs wounded with a mixture of venom and iodine solution, the dogs recovered, and the local appearances being watched with care, it was found that in one case only a little serous oozing took place, and in the other no secondary results could be observed. On the whole, therefore, the evidence is strongly in favor of the ability of iodine to diminish the local evils caused by venom, and, in so far, to lessen the general danger. If, too, a mixture of iodine and venom gives this as a result, it may be perfectly possible to modify a previous wound by the subsequent infiltration of neighboring tissues with iodine or tannic acid, and for this nothing can be more effectual than the mechanical means proposed by Dr. Brainard.

*Constitutional remedies.*—When defining the various fallacies which are to be avoided in studying the comparative value of remedies addressed to the cure of rattlesnake bites, I took occasion to define what was meant by an antidote, and so to correct the popular error, which limits its use to such substances as really neutralize the poison, whereas the derivation and actual meaning of the word more truly express the position of most remedies which possess any claim to the title, they being rather counteractive agents than anything more direct. Thus aconite enfeebles the heart, alcohol stimulates it, and is thus a physiological antidote to its effects.

A number of substances have at one or another period enjoyed the celebrity of being direct antidotes to venom, and even alcohol, which is certainly of use in these cases, has commonly been regarded as owing its value to some mysterious ability to neutralize the poison.

The remedies still in vogue as internal antidotes are ammonia, olive oil, cedron, guaco, arsenic, Bibron's antidote, (bromine,) and alcoholic stimulants. In studying these substances it will be proper to determine first, whether they directly neutralize, or rather alter the venom itself; and secondly, what power they possess when given internally. The first end may be attained by mixing the venom with an excess of the antidote, and injecting the mixture: if the venom acts us usual, the supposed antidote must be presumed to have no immediate influence on the venom.

The second method of study by the internal exhibition of the remedy will inform us as to whether it has the ability to counteract the effects of the venom. It may be pursued in two ways, by giving the antidote first, or by giving the antidote after the venom has been employed. Finally, it is absolutely necessary to give the venom in known amount by artificial subcuticular injection, rather than to trust to the uncertain plan of subjecting the animal used to the bite of a serpent.

*Ammonia.*—The volatile alkali, proposed by Jussieu in 1747, was closely studied by Fontana, who came to the conclusion that it was altogether valueless as a remedy in viper bite, whether used on the wound or internally, or in both ways at once. His experiments are open to criticism from the fact that he does not state how much ammonia he gave, or what effects it produced. On the whole, however, he makes out a strong case as against its antidotal value; but not even his high authority has prevented its frequent use, since his time, in India, Europe, and America. In our own country especially it has been regarded with much favor, and has received the support of numerous cases of rattlesnake bite ending happily under its use.* To know that a thousand cases of coryza got well after taking cubebs, gives us no strong conception of its saving power when once we learn that coryza does not kill; and in like

* Drake, West. Jour. Med. Phy. Sci., vol. i. p. 60; Miller, A. G., Boston Med. and Surg. Jour., vol. viii. p. 246, 1833; Moore, J., Am. Jour. Med. Sci., vol. i. p. 344, 1827.

manner when it becomes known that rattlesnake bites are really less fatal than they were once thought to be, we come to regard reports of their successful treatment, and limited mortality under this or that treatment, with some reasonable caution and reserve. To this fallacy, however, I have already alluded; it is well illustrated in most of the reports favorable to the use of ammonia. I have already shown, p. 294, that ammonia does not modify or affect at all the toxic activity of venom; when mingled with it and injected, the animals die as usual. If it has any value, it must be as a counter-active agent, and this, at first sight, seems not unlikely, when we recollect the symptoms of weakness which belong to cases of venom poisoning, and when we consider that ammonia is a stimulant.

It is scarcely requisite to give in detail the experiments upon the internal use of ammonia. Upon giving it to dogs which had been bitten, or to others into whose tissues I had injected venom, I failed to observe any marked stimulating effect, so completely was the remedy overmastered by the malady. In one case I believe that I actually destroyed a dog with the means which was meant to save him. On the whole, then, from my own results and those of Fontana and others, I am inclined to believe that the volatile alkali possesses no power over these injuries which does not belong in a far higher degree to other stimuli, which are usually more accessible.*

*Olive oil* has been used internally, both for viper bites and for those of the rattlesnake, and no remedy has been more favorably considered. The verdict of Fontana and the dictates of common sense are however against it, and it is quite impossible to perceive how in any way it could be of value.

*Cedron.*—The nut of the *simaba cedron*, and *guaco*, the root of *mikania guaco*, are two remedies which have been greatly praised, especially in South America. Cedron† is stated by M. Dumont (Aug. Dumeril‡ rep.) to be so perfect an antidote to viper venom that if freely given to an animal beforehand it becomes insusceptible to the effects of viper venom, and may then be bitten again and again with entire impunity. If this be correct, no better evidence of the value of this antidote can be offered,

* There is still another objection to ammonia, arising from the fact that it possesses the power of rendering the blood incoagulable, as Dr. Richardson, of London, has shown; and although the loss of coagulability arising from this agent and that which comes from venom poisoning are probably different, it should still have some weight in deciding our choice between ammonia and alcohol.

† Cedron is chemically a simple bitter, not unlike quassia.

‡ Notes Historique sur la Ménagerie des Reptiles du Muséum. Mém. du Muséum, vol. vii. p. 273.

and the only possible defect in the evidence might arise from the fact that the vipers were directly used, in place of known amounts of their venom. We shall see hereafter, that like powers have been ascribed to another antidote, which was found to fail under this test, applied with fitting precaution in my own laboratory. Late in the fall of 1860 I became possessed of a sufficient amount of cedron to test the above statements of M. Dumont, but unfortunately my snakes were torpid, and long captivity had so diminished their capacity to secrete venom that I was obliged to delay my examination of cedron until I should be more fortunately situated.

*Guaco*, vaunted by Vargas* and Chabert† as a prophylactic against snake bites, or rather their effects, must be a marvelous remedy if all said of it be true. I have no personal experience of its use.

*Arsenic*, unlike many of the supposed specifics against snake bites, certainly does not belong to the class of expectant remedies. It has been principally used in the East Indies, as the well-known Tanjore pill. This medicine is composed of arsenious acid, three East Indian roots, of which two are purgative, and one an active acro-narcotic, mixed with pepper, and the juice of the wild cotton plant. Each pill contains three-fourths of a grain of the arsenic. Two are given at once, and one at the close of an hour; no trifling dose of so potent a medicament. Russell, p. 65, examined this agent, and pronounced against its use; nor has it retained the celebrity which it once enjoyed. I have made no personal examination of its merits, nor indeed could I have procured the pill in question, which really contains other and active ingredients besides the arsenic.

*Bibron's antidote* has a very singular history, to which I have elsewhere called attention. It is thus described by my friend Prof. Hammond.‡

"Some four years since, Prince Paul of Wurtemburg, the celebrated naturalist, communicated to my friend, Mr. de Vesey, (Xantus,) the results of some experiments performed before the French Academy of Sciences by Professor Bibron, relative to an antidote to the venom of the rattlesnake. According to Prince Paul, Prof. Bibron allowed a rattlesnake to bite him in the lips, cheeks, etc., and by taking the antidote discovered by him, prevented all alarming symptoms, and, in fact, suffered no inconvenience therefrom. The antidote in question, as stated by Prince Paul, is prepared according to the following recipe: ℞.—Potasii iodid. grs. iv; hydrarg. chlorid. corrosid. grs. ij; bromini, ℥v. M. Ten drops of this mixture diluted with a tablespoonful or two of wine or brandy

---

* Semanario de agricultura yartes dirigido á los párrocas, vol. iv. p. 397, Madrid, 1798.

† Du Huaco et de ses vertus médicinales in, 8vo., 1853.

‡ Am. Journ. Med. Sci., No. lxix. p. 94, 1858.

constitute a dose, to be repeated if necessary. Prince Paul forwarded some of this medicine to Mr. De Vesey, who used it successfully in two cases of men bitten by rattlesnakes."

Prof. Hammond then proceeds to state two cases and certain experiments on animals. The first case was satisfactory as evidence, the second, which was reported by Dr. Coolidge, was less so, as Brainard's local treatment was also made use of.

The experiments were as follows: A young wolf was bitten by a very large rattlesnake. In thirty minutes, the symptoms being severe, six drops of the antidote were given. Relief occurred almost immediately, and recovery followed. Next day the same snake was allowed to bite the same wolf thrice. Before the antidote could be used, the animal fell and was apparently dead; the remedy poured into the throat remained there, but seemed to revive the animal for a time, when finally, at the twenty-seventh minute, death took place. The third case failed altogether, owing to the dog being unable to swallow when the antidote was given. In the last experiment, the snake having been used the day before, bit a dog in the jaw; his symptoms were not extreme, and he recovered perfectly after taking the antidote. Two of these cases, therefore, got well, and two died. Mr. De Vesey* (Xantus) reports a severe case of bite in a boy in whom the use of the antidote was followed by rapid amendment and permanent relief. Several experiments on dogs are also related, but the account given is wanting in details. He also states that dogs which had taken several doses of the antidote "*were for some time incapable of being infected by the venom of the rattlesnake.*" Dr. Heery† relates a case which was treated within five minutes, and promptly relieved. The information as to the bite was scarcely so full as might have been desired. Dr. Sabal‡ gave the antidote freely to six dogs, of whom two recovered.

The defects in the experiments above quoted must be plain to any one who has carefully followed my exposition of the fallacies which attend upon the direct use of the serpents in place of the injection of known quantities of their venom. Moreover, the reader will remember also that the suddenness of recovery is really a part of the natural phenomena of this mode of poisoning.

My own experiments with Bibron's antidote have been such, on the whole, as to lead me to regard it with considerable doubt. While expressing this opinion, I ought also to say that those who first used it in

---

* Am. Journ. of the Med. Sci., No. lxx. p. 375, 1858.

† Am. Journ. Med. Sci., No. lxxvi. p. 574, 1859.

‡ Savannah Journ. of Med., September, 1858.

this country were skilled observers, whose views are entitled to so much respect, that I shall still consider the question as somewhat unsettled until other observers have followed in my own track of research, employing the same caution which I have endeavored to preserve.

Before I became well acquainted with the peculiarities of the rattlesnake and its venom, I made the first series of observations which I shall now relate. Eight dogs were subjected to the fangs of rattlesnakes, which had been from three to five weeks in captivity, but had bitten nothing for a week. In all of the cases, the symptoms were serious; but of the eight only three died. A second set of dogs, eight in number, and resembling as nearly as possible the first set in weight and other circumstances, was selected for comparative study. After each of them was bitten, he was watched, and on the first appearance of grave symptoms, the bromine antidote was used in the manner about to be described: When no severe symptoms declared themselves within a reasonable period, a second snake was permitted to bite the animal; and again, upon the symptoms declaring themselves fully, the antidote was given as usual, although in somewhat smaller doses than those which I afterward employed. Of the eight dogs *three* recovered. So small a proportion, as compared with the recoveries of dogs bitten, but not treated, lends very little aid toward the solution of the question. I have selected, for full statement, three of the eight cases in which bromine was used:—

*Experiment.*—Black cur, weight 34 pounds, was bitten by both fangs $2\frac{1}{2}$ inches above and to the right of the anus; pulse, before the bite, a little irregular (a natural condition in the dog) and about 137; he yelled when bitten, and being set at liberty moved about uneasily. At the 15th minute, pulse 155, and weak; dog lying down, disinclined to move; muscles about tail twitching; he received 3 drops of the antidote in 10 of alcohol, and 4 ounces of water; the dose was given without the stomach tube and seemed to annoy him. 35th minute, swelling large and growing; pulse 120, feeble; respiration tremulous; when thoroughly aroused, he can walk, but seems inclined to lie down. 80th minute, pulse 130; swelling larger; took 4 drops of the antidote with water only; the pulse 124 to 130; no longer irregular, but feeble respiration as before. 119th minute, nausea and partial vomiting; appears stupefied; sensation still good. The presence of water seemed to excite in him the utmost dread; on its approach he rose and staggered backward, and fell as before; at the close of the third hour he took 5 drops of the antidote, and was then left until next day, when he was to all appearance well, and ate and drank greedily. The wound sloughed but little externally, although a large abscess formed beneath the skin. He gradually recovered from this, and experienced no secondary evils.

*Experiment.*—A mongrel terrier, weight $10\frac{1}{2}$ pounds, about 19 months old, was bitten under the jaw by one fang, and on the knee by two fangs. At the second minute he fell; with feeble pulse and much local quivering; took four drops of the antidote. At the 25th minute, micturated and passed solid stools; took six drops of antidote, shortly after which the quivering became general; the pulse more rapid and feeble; the breathing labored, and vomiting took place at the 60th minute. There was no odor of bromine in the matter ejected, and in general the speed with which this substance disappeared from the stomach was remarkable. Death took place at the 63d minute, without convulsions.

*Experiment.*—A large black terrier, weight 24 pounds, was bitten on the foreleg by one fang, and again by one fang on the inside of the hind leg, nine minutes later; the last wound bled about two ounces, and swelled but little; he sunk down almost immediately, making efforts to vomit. At the 18th minute, took four drops of the antidote, and again, three drops at the 32d minute; the respiration now became laborious and jerking, and the dog was unable to stand at all; five drops were now given, when he got up on his feet, coughed a little, and again lay down. At the 70th minute, another dose of four drops was given. At the 76th minute, the heart-beat imperceptible; respiration only occasional; involuntary evacuations; general fremitus; death.

In the remainder of my experiments I used known quantities of venom, which I injected; and the doses of venom employed were large and the dogs themselves of greater size. These observations were nine in number, and in all of them the amount of venom given was such as my experience taught must in all likelihood have been fatal were no antidote resorted to. In each of four cases the prophylactic power of the antidote was tested, and in the remainder this medicine was given *after* the venom had been injected.

*Experiment.*—Prophylactic power of bromine antidote. A brown bitch, weight 27 pounds, received through a stomach tube seven drops of the antidote in water; and in twenty minutes eight drops. Three minutes later, ten drops of rattlesnake venom was injected into the left fore shoulder; five minutes later, the local quivering was marked and the animal vomited some mucus and water, having neither smell nor color of bromine; pulse feeble and rapid. At the 74th minute, the animal was cold, and received five drops of the antidote; immediately after which she regurgitated a small amount of fluid into her mouth, but swallowed it again. The taste of the bromine seemed to annoy her, and she coughed considerably, and with much effort stood up and walked a few steps; this appearance of amendment was fallacious; she fell again, and so remained, until at the end of three hours and twenty minutes death occurred without convulsions.

*Experiment.*—A white cur dog, weight 24 pounds, took eight drops of the antidote at nine in the morning, and received no food up to a quarter to five P.M., when five drops of antidote were given in five ounces of water. Seventeen minutes later, ten drops were given as before; this dose appeared to annoy the dog, and in a few minutes he vomited; the matters expelled, water and mucus, smelled of bromine. At the 24th minute, five drops more were given, and again a slight attack of vomiting followed; but I did not think that he lost thus all of the bromine. At the 27th minute, I gave three drops of antidote, which were retained; and at once I injected ten drops of venom on the outside of the left fore shoulder. At 31st minute, made water freely. At 33d minute, very weak; stands with head drooping; quivering about wound conspicuous. 35th minute, urinated and defecated; lies down groaning. At 41st minute, gave three drops of antidote, when he rose up, moaned, and fell again; extremities cold; pulse feeble; respiration laborious and quivering. 42d minute, pupils dilating. 86th minute, general muscular quivering; wound much swollen; convulsive motions of head and jaw. Death at the close of an hour and forty-five minutes.

*Experiment.*—Mongrel dog, weight 23 pounds; took ten drops of Bibron's antidote, and in thirty minutes five drops more; forty-five minutes later, three drops more were given, and at the same time eight drops of venom were thrown into the left fore shoulder; in a few minutes the dog became extremely feeble, fell down, made unsuccessful efforts to vomit, and had a slight but general convulsive movement; at the close of an hour and twenty-five minutes, he was growing rapidly worse; when I gave five drops of antidote and left him. Sixteen hours after he was found dead and in complete rigor mortis.

*Experiment.*—The remaining case was differently treated. The dog received every night and morning seven drops of the bromine antidote during three days, and was then poisoned with seven drops of venom just after taking his last evening dose of the antidote. He died during the following night, the wound being found enormously swollen with fluid blood.

Five experiments were made with the same antidote, which I gave to dogs after injecting their tissues with venom. All of the dogs were large—none less than twenty-six pounds in weight—and all received the same dose of venom, about seven drops, and all were treated immediately with ten drops of antidote, and afterward with five-drop doses if they grew worse. If they mended, they were merely watched. Two of the five recovered; one of these being the smallest, the other the largest of the dogs. The remaining animals perished; one of them surviving thirty-one hours, and another three days.

The experiments here related, and the general results of the use of Bibron's antidote are, on the whole, so discouraging as to render it probable that it is in reality not more valuable than other agents which have once enjoyed an equal reputation. While expressing this opinion, founded chiefly on my own inquiries, I cannot help feeling that it is impossible to settle the question definitively without a further and larger experience in the form of human cases of venom poisoning, which should be studied with care by the light which I have endeavored to throw upon the subject—reference being had to the number of fang-marks, the size of the snake, etc.

*Alcoholic stimulus* is not so ancient a remedy in snake bites as most of the means already discussed. William Patterson, in 1791, after speaking of the effects of snake bites, advises the use of the Tanjore pill, and failing in this, Madeira wine, strengthened with brandy, and given in full doses.* In 1823 William Mayrant† reported cases of the use of alcoholic stimulus in snake bites. Dr. Atchison,‡ 1853, is the next authority advising its use; and finally, Dr. Alexander,§ in a brief essay, already alluded to, reports cases in which it was employed, and assigns to it a correct position as a physiological antidote, or counter-active agent. The three American authors last named mention the use of stimulants in these cases as if it were common, and in fact throughout this country the practical sagacity of physicians generally, and even of those who were not, seems to have perceived how plainly a stimulant was needed to correct the depression produced by venom. That the popular theory of the stimulus as a neutralizing antidote is incorrect, and that the remedy has been often abused in practice, admits of little doubt; but the general reliance placed upon it must, to some extent, be admitted as evidence in its favor, whenever other and more scientific proof of efficacy can be brought forward.

Besides the authors named, and a brief additional record by Dr. Burnett, there are no cases on record in which alcoholic stimulus was principally used. In examining the few cases alluded to, we are struck with the enormous amounts of stimulus which can be taken by a patient under the influence of venom, and indeed the very antagonism between the effects of the poison and that of the remedy which we may thence infer to exist, is of itself something in favor of the treatment alluded to. Many cases have been related to me, in which the patients took pints of

---

* Four Voyages into the Hottentot Country, and into Caffraria. 4to. 1791, London.

† Amer. Med. Recorder, vi. p. 619, 1823.

‡ Southern Journal of Med. and Phys. Sci., i. p. 47, 1853.

§ On the Use of Alcohol in Snake Bites; St. Louis Med. and Surg. Journal, xiii. 116, 1855.

brandy or whisky within a few hours; but I prefer to quote from Burnett* the following case, as sufficiently illustrative of my meaning:—

"Mr. B. was bitten just above the heel, when three-quarters of a mile from home. The usual symptoms of acute pain and large swelling immediately followed. He soon after complained of being blind and of having general pain, with the usual signs of prostration. The patient took one quart of brandy within an hour, but, although nauseated, he was not made drunk by this nor by a second quart, taken during the two ensuing hours."

In Dr. Atchison's† case a delicately-nurtured young lady, aged seventeen years, took within a few hours three pints of whisky, without the least intoxication being observable. The physician who recalls the cases of low fevers in which he has had occasion to give stimulants, with an eye to their effects less than to their amount, will not be surprised that, in the face of a deadly sedative, the system should acquire this tolerance of alcohol.

The manifest adaptation of stimulus to meet the indications in cases of rattlesnake bites is, then, a strong point in favor of this mode of treatment. If, forgetting the local cause, we were called on to observe a case of snake bite, and to note the enfeebled circulation, with pallor, nausea, and general prostration, we would almost by instinct resort to some stimulant, nor would our decision be incorrect. The early feebleness of the heart, and the depression of the nerve functions, so obvious to the eye, is not less notable when we come to measure, by more accurate means, the loss of cardiac energy, and the diminution of the intra-arterial pressure. For the details of the researches establishing these facts I must again refer to my former essay, contenting myself with the simple statement that the general result of my experimental inquiries led me even then to suspect that a rational treatment of venom poisoning could scarcely dispense with stimulants, at least in the early stages of the malady.

If, then, I am correct in my views, alcohol is to be looked upon as merely a counter-active agent; in a word, as a stimulus to be employed to buoy the patient over the prostration caused by venom poisoning. More than this it certainly is not, and those who have looked upon it as a direct chemical antidote, and as available for local treatment, need but to be told, to settle the matter, that a mixture of alcohol and venom is not less poisonous than the unmixed venom.

In conclusion, I ought perhaps to notice the argument against the use of stimulants, founded upon the fact that intoxicated persons have been bitten, and found no immunity in their condition. I have, however, discussed this question elsewhere, and I desire to state here only the simple

* Proc. Boston Soc. Nat. Hist., vol. iv. p. 315.

† Southern Journal of the Med. and Phys. Sci., vol. i. p. 47, 1853.

fact that, in the cases alluded to, no attempt was made to keep up the essential condition of stimulation. Furthermore, profound intoxication, where that has pre-existed, is not the condition which is to be desired, nor should we seek to do more, in using the alcohol treatment, than merely excite and sustain the flagging energies of the system, just as we would desire to do were the evil to be remedied an overdose of tartar emetic.

The following experiments were made in part during 1859 and 1860, and were broken in upon in the fall of the latter year, by circumstances which interfered with their completion. They are neither so numerous nor so varied as I desired to make them, but, in conjunction with what has already been urged, they appear to me to strengthen the popular notion in regard to the value of alcoholic stimulants in snake bites. The first two cases ended fatally, as follows:—

*Experiment.*—Dog of mixed breed, chiefly setter; weight 34 pounds. The animal standing, his pulse was 80, and but a little irregular. He was tied, as usual, to the leg of a table; pulse 86. The snakes had not been used for three weeks, and were very active. A large serpent bit the dog in the groin, upon which he howled fearfully, and made desperate efforts to escape. In 10 minutes, pulse 120. At 13 minutes he was bitten again by a snake, which struck him twice, in right groin and right shoulder, before he could be removed; 17½ minutes after first bite, pulse 134, feeble; 10th minute, dog howling, very weak, urine dribbling; 23d minute, gave 1½ʒ alcohol in 4 ounces of water, when at once he vomited a yellow bilious matter, and the pulse fell to 120; 26th minute, gave alcohol 1ʒ, as before, pulse 120, no longer irregular. At this time he lay down suddenly, and at the 28th minute took, with extreme difficulty, another drachm of the alcohol; 30th minute, general fremitus, pulse 120, but excessively feeble; 35th minute, took 2 drachms of alcohol, pulse 200, and feeble, breathing irregular; 48th minute, the dog was evidently dying, when he took 2 drachms of alcohol, and perished, with a slight general convulsion, 65 minutes after the first bite. The blood was diffluent, and did not coagulate on being kept for 24 hours. The colon contained dark fluid blood, and the peritoneum was dotted with ecchymoses.

*Experiment.*—In this case, a small cur, weight 10 pounds, was twice bitten in the leg by a large and active serpent. At the 5th minute he was feeble, pulse 120; before the bite, 116. 6th minute, gave 4 ounces of whisky, and repeated it at the 8th minute, when the heart was becoming more rapid and stronger. 10th minute, gave 1 ounce of whisky; pulse 128, and strong. At this time I was forced to leave him, and returning in an hour, I found him lying in a puddle of vomited matters; pulse 168, and feeble; I gave about 3ʒ whisky, but the dog ejected it at once, and, stretching himself, expired quietly.

The first of these cases was probably too freely poisoned for any hope of recovery. The second exhibited the constitutional effects of the stimulus, and might possibly have recovered.

*Experiment.*—Four efforts were made to test the prophylactic value of alcoholic stimulants. To each of four dogs, at different times, I gave two drachms of alcohol, in water; one became sleepy at once, the others were more or less excited. All were poisoned by the injection of 8 or 9 drops of venom; the injection being delayed an hour in the case of the dog, whom the alcohol stupefied. Under the influence of the venom, the exhilaration passed off rapidly, and in two the pulse rose and became feeble; while in the remainder it fell in force, but in number rested unchanged. The alcohol was now continued in each case, with one-drachm doses at intervals dictated by the symptoms. In the case of the dog specially alluded to above, the alcohol seemed to possess but little of its usual exciting power; his pulse changed less than the others, and in an hour and forty minutes he died convulsed, having taken in vain four ounces of alcohol. A second dog succumbed in three hours after the third dose; his heart failed to respond as before, and he sunk rapidly, but with his senses curiously clear, so that a few minutes before death, he followed the motions of a large fly hovering above him. The remaining pair both got well, and as the stimulus manifestly affected them, and as the dose of venom given was such as was likely to have killed, it seems fair to infer that the alcohol really aided them.

*Experiment.*—In two remaining cases, I gave whisky. One dog of rather small size took it with such freedom that, after being poisoned with 10 drops of venom, he swallowed within seven hours 18 ounces of this stimulus, not only without being injured, but with the most apparent good effects on his circulation and strength. The second dog experienced some good from the first dose which followed his poisoning, but he vomited so incessantly afterward as to baffle my efforts to keep up the use of the stimulus. I tried, finally, to excite him anew by ether inhalation, but the effort came too late, and he perished.

I am not disposed to lay too much stress on the above cases, but they taught me distinctly enough that stimulation was really of some use in venom poisoning, and I suspect that experience will hereafter bear me out in this conviction.

The disease caused by the venom is sometimes so prompt and terrible that it is impossible to rouse the system through the stomach, and this is doubly difficult when vomiting becomes one of the prominent symptoms of the general prostration. Under these circumstances, enemata of brandy may be used, and inhalations of hot alcohol or even of ether resorted to, in order to re-excite the flagging powers.

When called to a patient who has been bitten by a rattlesnake, the physician should at once ligate the limb with a *broad* band, as tightly as may be needed to check the circulation, while wherever it is possible cups should be also used immediately over the wound. The question of immediate excision or ablation of the part will be then determined by considerations already before the reader, (local treatment.) Setting these means aside, the iodine treatment, as limiting the local disease, may be then resorted to; but if, as is usually the case, there is no instrument at hand to make possible this treatment, incisions and the actual cautery are the final resort. Meanwhile, stimulus in some shape should be given, and when the excitement thus obtained is sufficient, the finger should be laid on the pulse and the band loosened. As the system becomes depressed, the ligature is once more to be drawn tighter, and, with continued use of stimulus, the economy prepared for another dose of the venom, which is thus to be antagonized little by little. Finally, it will be requisite to shift the band higher up the limb, to avoid the too great constriction of the damaged member. The further management of the case, with regard to stimulus, must be left to the physician, who will remember that in most cases of severe poisoning, he has to deal finally with a blood which has lost a part or the whole of its power to coagulate. He may find in the mineral acids, tonics, as quinine, and the continued use of stimulus, the necessary means for carrying his patient through the later stages of the malady.

I indulge the hope that the great interest of this subject may hereafter tempt physicians in this country to observe more minutely and record more fully such cases of snake bites as occur in their practice, observing them not as well-known maladies, but as instances of disease produced by animal poison, and of whose course and symptoms we know really but little.

